# Does relational capital matter to food and beverage SMEs’ resilience? The mediating role of environmental scanning

**DOI:** 10.3389/fpsyg.2022.1033837

**Published:** 2022-10-05

**Authors:** Yasmine YahiaMarzouk, Jiafei Jin

**Affiliations:** ^1^School of Management, Harbin Institute of Technology, Harbin, China; ^2^Faculty of Commerce, Damietta University, Damietta, Egypt

**Keywords:** relational capital, environmental scanning, organizational resilience, food and beverage SMEs, Egypt

## Abstract

The COVID-19 pandemic’s characteristics, including how quickly it spread, and the emergence of new virus variations, raise serious questions about the pandemic’s potential repercussions and complications in the food and beverage industry, among other industries. The global COVID-19 pandemic highlights the pressing need to reconsider how we manufacture and market food and beverage goods. During the epidemic, SMEs must build organizational resilience (OR) in order to recover economically, socially, and communally. Relational capital (RC) is a crucial factor that can be deployed by SMEs to acquire the resources existing in the external networks to adapt to disturbances; however, the impact of RC on the resilience of Egyptian food and beverage SMEs is under-examined. Additionally, it is unclear how RC promotes organizational resilience. In this regard, we argue that social interactions and regular communication can let an SME and its business partners exchange information and best practices, thereby enabling it to immediately establish backup plans to deal with the disruption. In order to achieve our aim, we investigated how relational capital affected organizational resilience both directly and indirectly through environmental scanning, utilizing partial least squares structural equation modeling (PLS–SEM).The results from 217 Egyptian food and beverage SMEs demonstrate that relational capital directly and positively affected organizational resilience. Besides, the RC–OR relationship is partially mediated by environmental scanning. Our current study, therefore, adds to the extant literature through being one of the few studies to address the topics of relational capital and resilience altogether during crisis times within a developing country, an issue that has not been sufficiently investigated in exiting literature. Moreover, our current study is the first one to empirically investigate the role of relational capital in facilitating environmental scanning activities of SMEs to ultimately foster their resilience. Our results, thus, prove that a large amount of information relevant for recovery are inherent within an SME’s external relations network. We provide implications for theory and practice.

## Introduction

Globally, the COVID-19 pandemic is multidimensional; at the outset it is a health crisis, but at its core it is an economic crisis that affected all sizes and types of manufacturing industries, including SMEs and food and beverage industry, making it a triple shock (Erokhin and Gao, 2020; Mouloudj et al., 2020). When it comes to the local economy, the crisis influenced the growth rate of the GDP, which was reduced to 3.6% for the fiscal year 2019–2020. SMEs include 1.7 million businesses, account for 95% of all industrial enterprises, create 34% of all revenue generated by Egyptian businesses, and employ 75% of the total workforce. SMEs represent 13% and 46% of Egyptian industrial enterprises, respectively (Zgheib, 2019).

The food system in Egypt generates 24.5% of Egypt’s GDP and 23.2% of its labor value added. SMEs dominate the food and beverage industry in Egypt (UNIDO, 2020). The food and beverage sector ranks first in terms of manufacturing employment, second in terms of manufacturing value added, and third in terms of manufacturing exports in Egypt (UNIDO, 2020).

Almost all countries have experienced interruptions in the shipment and distribution of food and beverages due to COVID-19, which has had a number of detrimental repercussions. The numerous preventive restrictions associated with the epidemic, such as lockdowns, remote work, social distancing, etc., severely impacted the food and beverage supply chains globally during the pandemic and exposed their vulnerability to shocks and crises (Mukhamedjanova, 2020; Hassen et al., 2022). Additionally, a new issue relates to the availability of workforce in the food and beverage industry, since significant labor limitations have been emerged (Martínez-Franco et al., 2021). Generally speaking, the pandemic has also changed how households in various parts of the world consume food and beverages (Dudek and ´Spiewak, 2022; Hoteit et al., 2022).

Locally, the report of the United Nations (UN) examined the effects of the COVID-19 epidemic on the food industry of Egypt and made recommendations to support the sector’s adjustment, recovery, and transformation. The study’s key recommendations included advice and information (e.g., on contingency measures, external opportunities, quality, and safety; UNIDO, 2020). Besides, the Egyptian food manufacturer and industry associations surveyed in this study acknowledged that to minimize the sector’s sensitivity to external shocks (e.g., the COVID-19 crisis), it is necessary to diversify suppliers and build strong relationships with different parries (UNIDO, 2020).

Despite the recommendations provided by the UNIDO’s study regarding the importance of information about market opportunities, contingency measures, safety, and quality and the diversification of suppliers and building of strong relationships with different stakeholders for survival of Egyptian food and beverage SMEs, no study until now has empirically investigated the effects of information gathering and relationships with external parties on the resilience of those SMEs. Therefore, to fill this knowledge gap, we investigate the effects of relational capital along with environmental scanning on organizational resilience of Egyptian food and beverage SMEs amidst the COVID-19 pandemic.

Resilience can be described from a managerial perspective as the capacity of individuals or organizations to successfully recuperate and withstand challenging circumstances, demanding conditions, or unpredictable events. This characteristic, which is demanded by organizations working in complicated, dynamic, and fluctuating circumstances, enables adaptability to crises and periods of social, economic, and/or political turmoil sans substantially compromising the effectiveness of an organization (Annarelli et al., 2022). In addition to attempting to comprehend what resilience is, research on organizational resilience also aims to explain how and why certain organizations are more resilient than others (Duchek, 2020). Determining the internal and external variables that are more significant and the role played by external variables in supporting organizational resilience still unclear (Jia et al., 2020). Cooperation, relationship behavior, and partnership are examples of external variables that have attracted little attention in previous research (Jia et al., 2020).

The capacity of an organization to foresee external environmental changes and consider this amidst formulating its strategy is a key component of its ability to survive (Aldehayyat, 2015). The rising interest in environmental scanning, a method of acquiring pertinent information and transforming it into knowledge, which can be deployed in operating a business, is due to the necessity to recognize and adapt to external changes in the external environment (Aldehayyat, 2015).

Despite the growing number of empirical research that have investigated ES activities in enterprises all over the world (Jorosi, 2008; Wong and Hung, 2012; Aldehayyat, 2015), this filed remains a highly untapped one within developing nations generally and particularly in Egypt. Moreover, the empirical studies investigating the factors that can underpin the environmental scanning activities are still largely lacking.

Additionally, SMEs typically lack crucial resources, which prevents them from having the infrastructure necessary for thorough data collection and searching, which in turn limits their capacity for scanning (i.e., the ability to obtain all relevant environmental information; Tang, 2016). Instead, we argue that having trustworthy relationships with business partners (i.e., strong relational capital) can facilitate the SMEs’ environmental scanning activities through providing and exchanging the relevant information among all relevant parties. No study has yet empirically examined the effect of relational capital on environmental scanning.

In addition, Bhamra et al. (2011) noted that it is necessary to investigate how SMEs might build resilient features *via* collaboration of networks in their assessment of the resilience literature. Due to resource limitations that larger companies typically do not suffer (e.g., liquidity, getting finance, and a greater difficult policy climate), SMEs may be more vulnerable to shocks (Ozanne et al., 2022). Additionally, SMEs may find it challenging to anticipate and prepare for disruptive occurrences because of their size. (Ozanne et al., 2022). Their vulnerability to disturbances may therefore be greater, posing a challenge to their mitigation, response, and recuperation strategies (Ozanne et al., 2022). A vital resource that SMEs can use to respond to disturbances is relational capital. This capital is inherent in relations with external business parties (Ozanne et al., 2022).

The review of the literature conducted by Walecka (2021) on relational capital and crises in enterprises revealed that both of these topics have not been sufficiently investigated. It is important to emphasize that although there is a wealth of research in the field of crisis management regarding determinants of enterprises’ resilience to crises; it rarely emphasizes the significance of RC as a means in anti-crisis strategies (Walecka, 2021). Based on the observed research gaps, the authors conclude that the relational capital of a firm is a significant, albeit overlooked, factor influencing environmental scanning activities and a firm’s resilience to crisis occurrences. Besides, previous studies in the field of ES are either just theoretical ones recommending the importance of studying the role of ES in enhancing OR (e.g., Vakilzadeh and Haase, 2021; Thomas and Suresh, 2022), or empirical studies investigating its direct and indirect impacts on resilience (e.g., YahiaMarzouk and Jin, 2022a,b), or investigating the consequences of ES (e.g., Fathi et al., 2021; Blaique et al., 2022). Accordingly, we noticed that most of ES studies are ignoring the factors that can facilitate the scanning mission of managers. No previous study has empirically investigated the direct and indirect effects of relational capital on organizational resilience through environmental scanning within the developing context of Egypt during the COVID-19 pandemic. As a result, this specific line of research has been recognized as vital and interesting from a scientific perspective. Therefore, this study recognizes the value of a strong relational capital in developing crisis resilience through environmental scanning. Hence, the key aim of the current study is to investigate how relational capital is related to environmental scanning and organizational resilience. Accordingly, the questions arising in the current study are as follows:

1. What are the direct effects of relational capital on environmental scanning and organizational resilience?
2. To what extent does environmental scanning mediate the relationship between relational capital and organizational resilience of Egyptian food and beverage SMEs?

The current study investigates this issue within Egyptian food and beverage SMEs because food and beverage products are regarded as essential consumer products and production in Egypt depends heavily on locally produced goods (Breisinger et al., 2020). Although the COVID-19 pandemic influenced the food supply chains’ smooth operations and resulted in food insecurity conditions in numerous countries (Memon et al., 2021), the effects on food and beverage sectors are less severe than elsewhere in the Egyptian economy (Breisinger et al., 2020). Besides, losses in food processing are relatively low. For example, recent reports show that food and beverage products have seen an increase in demand and that local processing has been increasing to substitute for the decrease in imports of processed food and beverage items during the pandemic. Therefore, it was expected that the Egyptian food and beverage sector could see an increase of 10% because of the COVID-19 crisis (Breisinger et al., 2020).

Despite the importance of the food and beverage sector to the Egyptian economy, the resilience this sector showed during the pandemic, which was indicated by a 10% increase in production, and the variety of research that has been conducted on the effect of the epidemic on different supply chains, no study has been conducted regarding the responses of the food and beverage SMES in African countries during the pandemic. More specifically, no study until now has tried to empirically investigate the factors contributing to Egyptian food and beverage SMEs’ resilience to the pandemic. Therefore, we contribute to the extant literature and to the Egyptian food and beverage sector by highlighting that food and beverage manufacturers may even benefit from the COVID-19 pandemic if they are able to engage in strong external relationships to provide them with the materials, resources, and information relevant for survival.

Our current study, therefore, contributes to extant literature by (1) clarifying the role of relational capital on resilience during COVID-19 crisis within the developing market context of Egypt, an issue that has not been sufficiently investigated in exiting literature; (2) being the first study to empirically investigate the impact of relational capital on environmental scanning; and (3) introducing new insights that explain how SMEs can benefit from the large amount of information and resources inherent within their external relations network to survive.

## Literature review and hypotheses development

### Key concepts and theories

#### Relational capital

Relationships are the basis of the modern world. Every firm transfers both tangible and intangible assets from and to the business environment since it is an open system. Every firm must establish diverse networks of relationships with clients, suppliers, and rival businesses in order to remain competitive (Walecka, 2018). In this regard, the value, which is generated and preserved through having, fostering, and maintaining good relations is referred to as relational capital. Relational capital provides assets through the development and deployment of relationships, which turn relationships into resources for achieving both individual and collective goals (Blatt, 2009). The notion of relational capital was originally publicly disclosed by Granovetter (1985), who defined it as a resource that is present in interactions between individuals and organizations. Later, Morgan (1996) undertook more thorough investigations of relational capital, highlighting the fact that it is a non-market resource that resides outside the organization and has the potential to provide value to it.

Relational capital is defined as the sum of relationships among a firm and its key business parties and is operationally defined *via* factors such as image, customer satisfaction and loyalty, connections with suppliers, environmental activities, commercial power, negotiation skills with financial enterprises, and so forth (Walecka, 2021). RC is described as the relationship a firm develops with its internal and external stakeholders, including its clients, suppliers, staff, and partners in strategic alliances (Ordonez de Pablos, 2003). Organizations are connected with external stakeholders, such as customers and suppliers, *via* these relationships, and RC plays a crucial role in developing relationships among organizations and their stakeholders (Håkansson and Johanson, 1992). RC involves strong understanding and cooperation among partners, higher levers of trust, and particularly stronger relations between the organization and its strategic partners (Ahmed et al., 2022).

One of the most crucial dimensions of intellectual capital is relational capital (Rooss and Roos, 1997) and quickly sparked attention as an independent field of research and the topic has been addressed in publications by several researchers (e.g., Zanda and Lacchini, 1991; Nahapiet and Ghoshal, 1998; Ulrich, 1998; Costabile, 2001). In the management literature, relational capital is defined in numerous ways. For instance, it is defined as the total of resources that are actual or potential and embedded in, drawn from, or emerging from external relational networks (Cousins et al., 2006). RC also refers to consistency between and within firms in terms of notions of identity, belief, responsibility, and obligation (Liao, 2018). It refers to the depth of the partnership, wherein trust, friendship, respect, and reciprocity are ingrained and strengthened as a result of businesses’ recurrent business transactions with their stakeholders (Nahapiet and Ghoshal, 1998; Tsai and Ghoshal, 1998). Relational capital is the capital that aids businesses in acquiring resources *via* networks of high quality; yet, network kinds, densities, sizes, and other elements also have an impact on businesses’ capacities to obtain resources (Liao, 2018).

Although there are numerous definitions of RC in the literature, there is not agreement among researchers over a single definition. For the purposes of this study, we therefore assume that relational capital is the sum all of the relationships of an organization with its environment, particularly those with clients, rival businesses, suppliers, and other strategic partners as well as with financial institutions, local government entities, organizations involved in the labor market, and other stakeholders (Walecka and Zelek, 2017).

High relational capital fosters open communication, transparent behavior, mutual trust, and exchange of necessary and valuable resources and information among business parties, which can help SMEs come up with innovative solutions to problems after a crisis (Ozanne et al., 2022).

Particularly, due to reliable and mutually beneficial relations, business partners are more inclined to work collaboratively amid unpredictable events in terms of lead times, expenses, and credit (Jia et al., 2020). Relational capital is crucial for a firm’s ability to respond to crises (Gittell et al., 2006; Ahangama et al., 2019) because it makes it easier to build strategies for dealing with unexpected developments and to focus organizational efforts on finding win-win solutions (Ortiz-de-Mandojana and Bansal, 2016).

#### Environmental scanning

Environmental scanning, according to Aguilar (1967), is the activity of looking for and gathering information on occurrences, developments, and shifts that are occurring outside of the firm to shape its future course of action. Accordingly, environmental scanning, which focuses on gathering and using information on external events and trends, is regarded as a crucial part of the strategic planning process (Aldehayyat, 2015). Lester and Waters (1989) described it as a managerial tool, which uses information about the environment to promote decision-making through the collection, evaluation, and use of information.

A technique for strategic foresight is environmental scanning. Strategic foresight aims to account for unpredictable decision-making processes and limit the unknown area (Ratcliffe, 2006). Environmental scanning is one of the strategic foresight techniques that helps firms become alert to their environments and effectively seek out and capture chances that are missed by competitors in extremely volatile circumstances (Sarpong and Maclean, 2011). Additionally, research have revealed that some firms employ strategic foresight techniques to strengthen their resilience to environmental disruptions (Madjdi and Huesig, 2011).

Environmental scanning is used to spot and monitor existing and emerging trends that present chancy opportunities and present obstacles to an organization’s long-term development (Costa and Teare, 2000; Jogaratnam and Law, 2006). Business decision-makers can recognize and foresee environmental change through using environmental scanning to understand the external environment’s occurrences (Coulter, 2013). Costa (1995) asserts that scanning enhances an organization’s capacity to manage speedily changing environments by capitalizing on opportunities early, delivering an early warning of implementation issues, increasing an organization’s awareness of the changing needs of its clients, and enhancing an organization’s reputation with the general public by demonstrating that it is attentive and adaptable to the environment.

#### Organizational resilience

The field of ecology was the first field to give rise to the notion of resilience, which is described as “the capacity of a system to absorb change, while persisting development in the original state subject to disturbances and changing conditions” (Holling, 1973; 17). The studies on organizational resilience that follow become multi-level and interdisciplinary, including everything from natural sciences like ecology and engineering management to social sciences like economics, management of organizations, strategic management, and supply chains (Saad et al., 2021).

Organizational resilience was originally discussed in the context of organizational management research by Meyer and Rowan (1977). The interest of researchers in organizational resilience has increased recently but there is a lack of consensus on its conceptualization (Dhoopar et al., 2022). Typically, the concept of resilience in management and organizational research literature refers to an organization’s capability to survive while facing unanticipated changes (Hall and Prayag, 2018). Kantur and Iseri-Say (2012; 763) defined OR as “an organization’s capability for turning adverse conditions into an organizational opportunity, positive attitude of ‘bouncing back’ and a relatively agile deportment.” Similarly, organizational resilience refers to “the incremental capacity of an organization to anticipate and adjust to the environment” (Jia et al., 2020: 2). Organizational resilience is a dynamic quality that organizations may or may not possess. It is generated through a combination of capacities that result from recognizing and addressing destructive tendencies in response to disruptions (Sutcliffe and Vogus, 2003; Ortiz-de-Mandojana and Bansal, 2016). Organizational resilience is generally understood to be the capacity of organizations to effectively absorb, adjust to, and finally benefit from disruptions that may jeopardize their survival, despite the fact that academics have not yet reached an agreement on defining this concept (Williams et al., 2017).

Organizational resilience is crucial for businesses’ success in tumultuous times since it enables them to adapt to a variety of interruptions, from unpleasant incidents to global catastrophes (McCann et al., 2009). To handle the continual environmental pressures, organizations must constantly work to increase their organizational resilience. Nevertheless, for SMEs, it is still difficult to identify the basic factors, practices, and resources that support organizational resilience (Sanchez-Garcıa et al., 2020). Therefore, research into the identification of the practices and behaviors required for businesses to be more resilient is becoming a growing emphasis of management literature (Lai et al., 2016).

It is necessary for organizations to concentrate on developing resilience capacities since it enables the organization to respond to the unpredicted events and benefit from them instead of letting such crises negatively influence the organization’s survival (Gupta et al., 2022). Organizational resilience not only enables the organization to effectively respond to changes, but also it improves the sense of well-being and motivates organizational members to make sense of change rapidly along with helping them to maintain their performance levels. During the COVID-19 crisis, it is important for the organizations to be resilient enough to fight such a massive scale crisis (Dhoopar et al., 2022).

Although there is a rising interest in organizational resilience and the several dimensions that different authors have developed for it, there is not an agreed upon measure to quantify it (Kantur and Iseri-Say, 2015). In organizational and strategic management studies, the concept of organizational resilience is frequently employed. While qualitative and theoretical research are developing, quantitative investigations are moving more slowly. This is because of the lack of a valid and reliable measurement scale in the extant studies (Vogus and Sutcliffe, 2007). Accordingly, we will rely on Kantur and Iseri-Say’s (2015) scale of OR. Instead of measuring the extent to which the variables that contribute to resilience are present, this scale measures the organization’s level of resilience. Therefore, the dimensions of OR according to Kantur and Iseri-Say (2015) include robustness, which assesses a firm’s capacity to resist and bounce back from challenging situations; agility, which assesses a firm’s ability to act quickly; and integrity, which measures the cohesiveness among organizational staff.

### Conceptual framework and hypotheses development

The resource-based view argues that all of a firm’s assets, capabilities, organizational processes, information, and knowledge are its resources. These resources are used by businesses to develop and carry out their strategies (Wernerfelt, 1984; Barney, 1991). A firm cannot be completely self or possess all of the resources required for development. There are many scarce resources needed for a firm to survive in the external environment (Salancik and Pfeffer, 1978). As a result, a firm should rely on the external business environment for the resources it needs to survive, adapt, and prosper. In this regard, the best method of obtaining these resources is through the utilization of relational capital (Liao et al., 2018).

Relational capital is crucial for a firm’s ability to respond to crises (Gittell et al., 2006; Ahangama et al., 2019) because it makes it easier to build strategies for dealing with unexpected developments and to direct firm’s efforts toward finding mutually beneficial solutions (Ortiz-de-Mandojana and Bansal, 2016). Jia et al. (2020) discovered that networks and resources enterprises have access to because of their linkages help organizations create organizational resilience.

Strong relational capital in a network is exemplified by shared values such as mutual trust, respect, commitment, reciprocity, and devotion to one another (Nahapiet and Ghoshal, 1998). In these relationships, organizations frequently work together to support business recovery (Prasad et al., 2015). Organizations can leverage the resources of their business partners to promote organizational resilience by developing partnerships based on mutual trust, commitment, reciprocity, and respect (Jia et al., 2020). Additionally, when businesses are heavily reliant on external resources and incapable of running their operations effectively on their own, their relationship networks will be extremely helpful to them in adapting to the environment (Fang et al., 2016).

Trust-based (relational) interactions foster a sense of commitment among business partners, enabling firms to respond with effective business operations (Ozanne et al., 2022). Additionally, it makes it easier to collaborate across departments, reducing risk and speeding up the recovery (Polyviou et al., 2020; Ozanne et al., 2022). In fact, relational capital supports SMEs’ adaptation to environmental changes by assisting them in comprehending and utilizing pertinent information about patterns and events common in external environments (Liao, 2018).

Each organization transfers both tangible and intangible resources to and from the business environment as an open system. It must create various networks of links with its stakeholders, such as clients, suppliers, or rivals, in order to survive. A firm’s likelihood of adapting and surviving is increased by having good relationships with the environment, which helps it better comprehend the demands of its environment (Walecka, 2021). Despite the dynamic and discontinuous nature of the environment, businesses must not only adapt to it but also co-create with it (Walecka, 2021). As a result, businesses develop relationships with the environment that enable them to produce added value (Johnson and Selnes, 2004).

One of the techniques used to lessen environmental uncertainty is increasingly building partnerships with various stakeholder groups and strengthening relational capital within an organization. An organization’s flexibility is greatly increased by its capacity to forge strong relationships with various stakeholders, which substantially boosts its resilience to crises (Walecka, 2021). A creative reaction is primarily made possible by structural linkages strengthened by relational capital (Sienkiewicz-Małyjurek, 2022).

To respond swiftly to environmental changes and fluctuating market demands, organizations can make use of their RC and the opportunities for trust-based collaboration that it offers. RC could therefore aid businesses in surviving in a competitive climate. When knowledge is essential for achieving resilience, social networks between various stakeholders along the value chain may increase market awareness and offer businesses an efficient means to participate in knowledge exchange activities (Ahmed et al., 2022).

In times of crisis, RC seems crucial for surviving. In this regard, Walecka (2021) discovered that the organizations surveyed had stronger crisis resilience due to their relationships with external stakeholders, which helped them avoid numerous crisis scenarios. The stakeholders in the surveyed companies have successfully mitigated crises because of their positive attitudes. This circumstance also applies to the current COVID-19 crisis. Accordingly, our study formulates the following hypothesis:

*Hypothesis 1:* Relational capital significantly and positively affects organizational resilience.

Environmental scanning relies on a combination of two information sources. Such sources include both internal and external information about employees, managers, and decision-makers as well as information about customers, competitors, and governmental and regulatory bodies, respectively (Aldehayyat, 2015). In this regard, earlier research suggests that RC makes it possible for organizations to communicate with other stakeholders and gather more information on the external environment (Fang et al., 2016). As a result, we can expect that RC will be able to facilitate and improve ES activities because strong, trustworthy bonds encourage sharing of information among a firm and its business partners (Jia et al., 2020).

Comparing SMEs’ environmental scanning behavior to that of large corporations, some differences may exist (Aldehayyat, 2015). For instance, in SMEs, the owner-managers themselves are typically in charge of ES activities. However, SMEs often do not have the necessary infrastructure to gather and search for information effectively. SMEs instead rely more on information gained through networks and other types of associations (Liao et al., 2018).

A higher level of RC enables businesses to share ideas and collaborate with other businesses, communities, and governmental agencies out of a sense of trust and mutual benefit to gather important information. RC is particularly significant for businesses that depend more heavily on external resources, such as those in the food and beverage industry (Fang et al., 2016). Information sharing achieved through RC can increase efficiency and decrease costs for production (Cousins et al., 2006).

ES activities can be facilitated by strengthening a network of trust and support between a firm and its business partners. This is because when there is trust, business partners are less hesitant to provide information or raise potential environmental concerns (Gu, 2018). By facilitating a steady flow of information from numerous external sources, RC can make it easier to carry out ES activities (Blyler and Coff, 2003).

The implementation of ES activities can be facilitated by a climate of trust among business partners, thus enhancing an enterprise’s capacity to recognize threats and opportunities (Fainshmidt and Frazier, 2017). In order to explore and take advantage of environmental opportunities, actors might communicate tacit knowledge through a dense network with external partners, which fosters learning (Ozanne et al., 2022). In order to provide valuable client offerings, RC gives SMEs access to crucial information. This is particularly advantageous in disruptive contexts (Ozanne et al., 2022).

Moreover, Zahoor and Gerged (2021) asserted that alliances with different parties including clients, suppliers and/or rivals give SMEs access to information and resources that can be used to develop solutions for social and environmental problems. Specifically, RC is proven to be useful in facilitating the exchange of information (Zahoor and Gerged, 2021), which has the potential to promote ES activities.

Relational capital can be particularly crucial for providing useful information by fostering trust and formulating environmental policies among business partners (Kohtamäki et al., 2012). In other words, RC might inspire creative approaches for the alliance partners to integrate environmental information (Zahoor and Gerged, 2021). Because RC exhibits increased levels of trust and respect among business parties, these social relations generate better channels for the exchange of environmental information (Zahoor and Gerged, 2021), thereby facilitating the activities of ES. Accordingly, our study formulates the following hypothesis:

*Hypothesis 2:* Relational capital significantly and positively affects environmental scanning.

Environmental scanning gives top management the information they need to make decisions that will give the firm a strategic advantage to respond to the fluctuating environment (Aldehayyat, 2015). The literature has emphasized the importance of closely observing both internal and external environments to deal with future issues (Burnard et al., 2018). Environmental scanning as well as other foresight techniques are meant to increase the adaptability and flexibility of enterprises by enabling the early detection of significant events and occurrences. Foresight units provide up-to-date information on pertinent events and alterations to other organizational divisions, enabling them to promptly adapt their tactics (Haarhaus and Liening, 2020).

Additionally, organizational resilience is defined as a company’s ability to recognize, address, and avert dysfunctional occurrences as well as react successfully with unexpected events (Ortiz-de-Mandojana and Bansal, 2016). This implies that OR begins with spotting and sensing adverse occurrences taking place externally. Such a function of sensing is the primary function of ES. Organizations need information about relevant events and changes. In this vein, ES is typically utilized to determine and comprehend dysfunctional occurrences in the external environment; hence allowing it to speedily direct resources towards novel action courses in response to environmental changes. Consequently, the respective strategies can be quickly adjusted (Haarhaus and Liening, 2020).

For proactive, constant modification and adaptation that increases an organization’s capacity for resilience, crisis awareness and an understanding of unfavorable environmental shifts are essential. Therefore, managers should routinely arrange forward-looking discussions with the organization’s internal and external parties to stimulate anticipation of the future and motivate their organizations to adapt to changes (Lv et al., 2018). *Via* ES, an enterprise becomes able to comprehend prospective challenges that may arise in the future (Paraskevas and Quek, 2019). Being aware of such warning signals in advance enables the organization to prepare mechanisms to absorb shocks beforehand, thereby avoiding or mitigating future challenges (Carmeli and Markman, 2011).

Similar to this, Hillmann and Guenther (2021) stressed the importance of alertness, sense-making, and prediction of hazards and potential developments in order to lessen organizations’ susceptibility and increase awareness, thereby strengthening their resilience (Hillmann and Guenther, 2021). By improving their employees’ perception, tracking, and response to environmental changes, organizations can respond promptly to environmental shifts. In a similar vein, managers need to intentionally position their enterprises to be among the first to discover and acquire information about external environmental events, technologies, and industry in order to quickly adapt to environmental changes and improve their resilience (Akpan et al., 2021).

Vakilzadeh and Haase (2021) discussed the role of ES as a building block of resilience and noted that some prior studies highlighted the role of sense making to anticipate change and adapt. Similarly, Tisch and Galbreath (2018) referred to the importance of the early identification of environmental signals by analyzing how New Zealand farmers cope with expected extreme weather-based calamities. The farmers go beyond merely comprehending the situation and translate these signals into timely decisions, allowing them to predict and prepare for adverse events. The ability of OR to recover from the disaster by raising environmental awareness to cope with disturbances and interruptions was also stressed by Pratono (2022).

Gathering environmental information provide a real time early warning of impending threats, thereby facilitating adaptation to them (Barasa et al., 2018). Resilience basically involves the anticipation of and response to the threats of external crises and shocks. This means that anticipation, which can be achieved through ES, is a main factor contributing to resilience. Organizations need to concentrate on monitoring what is going on to observe if anything changes that might endanger their capacities to conduct current or planned operations (Thomas and Suresh, 2022).

Environmental scanning helps businesses identify external developments and trends and identify the skills needed to successfully adapt and become more resilient (Beal, 2000; Castanias and Helfat, 2001). As a result, we believe that an organization’s capacity to foresee change and its readiness to respond are key components of resilience. In this way, environmental scanning is a tool for identifying potential dangers and opportunities in the external environment. Accordingly, our study formulates the following hypothesis:

*Hypothesis 3:* Environmental scanning significantly and positively affects organizational resilience.

By combining H1, H2 and H3, we argue that ES mediates the relationship between RC and OR. This hypothesis is built on the notion that RC enhances ES, and that ES is positively related to OR. Amidst an unpredictable disruption, business partners are expected to provide resources and information that can facilitate SMES’ recovery (Prasad et al., 2015).

Sharing information between network participants, according to Wang et al. (2018), reduces the likelihood of future disturbances. By adding slack to the supply chain, network participants can anticipate changes in a proactive manner (with the aid of an effective monitoring system) and adopt solid responses (Jia et al., 2020). As a result, sharing crucial information with business partners requires cooperation (Blackhurst et al., 2011; Wieland and Wallenburg, 2013). Sharing information makes risks that can be minimized more visible, which strengthens the firm’s capacity for resilience (Durach and Machuca, 2018).

According to Ozanne et al. (2022), social interactions and regular communication let the organization and its business partners exchange information, knowledge, and best practices. This enables them to immediately address the impact and establish backup plans to deal with the disruption. Accordingly, our study formulates the following hypothesis:

*Hypothesis 4:* Environmental scanning mediates the relationship between relational capital and organizational resilience.

**Figure 1:**
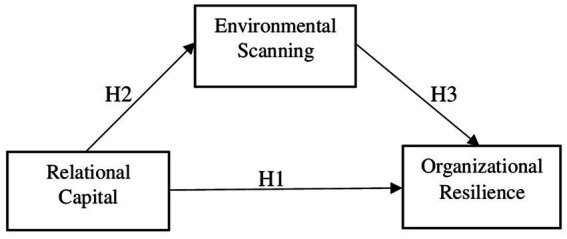
Conceptual model.

The conceptual model (Figure 1) depicts the relationships between study variables.

## Materials and methods

### Sample and data collection

This study aims to understand the effect of relational capital on organizational resilience and the mediating role of environmental scanning in the RC–OR relationship within Egyptian food and beverage SMEs. A cross-sectional questionnaire-based survey was used to gather data amid the COVID-19 epidemic in order to test the suggested study model. The sample for the current study was drawn from the food and beverage sector in Egypt and included respondents representing SMEs.

The key respondents of our study are chief executive officers (CEOs). We believe that the CEOs are suitable respondents due to three reasons. First, CEOs are regarded as the single most knowledgeable and reliable information providers in SME research. (Lechner et al., 2006, p. 525). Second, the management, values, and decision-making of SMEs are particularly impacted by CEOs (Eggers et al., 2013). Third, in SMEs, the CEOs are typically in charge of ES activities (Liao et al., 2018). Consequently, CEOs are frequently used in SMEs research as key informants (e.g., Amankwah-Amoah et al., 2019; Danso et al., 2019).

Five hundred SMEs in the food and beverage industry were chosen at random and given a total of 500 questionnaires. Random sampling premises that any case of the population possesses an equal chance of being chosen. Random selection of participants from a population is referred to as “random sampling.” Consequently, a sample clearly reflects the entire population (Mackey and Gass, 2012). Most researchers favor random samples as they increase external validity, remove the bias of the researcher in selecting the sample, and improve generalizing from the sample to the whole population (Begum et al., 2021).

To improve the CEOs’ inclination to participate in this survey, we clarified our research goals to them. In addition, we ensured the respondents’ privacy by guaranteeing not to share their information in the questionnaire cover letter while outlining the aim of the study. The participating CEOs were contacted 2 weeks after the surveys were distributed and reminded to finish the survey in an effort to increase the questionnaire return rate. A total of 253 CEO surveys were returned back, 36 of which were not complete and therefore not included in the ultimate sample. Finally, 217 valid surveys were included in the sample, with an effective response rate of about 44%. Table 1 illustrates specific demographic characteristics of the sample.

**Table 1:** The demographic characteristics of the sample (*n* = 217).

Respondents	Proportion (%)	SME category	Proportion (%)
Gender		Size	
Male	91.24	Lower than 10 employees	19.82
Female	8.76	10–50 employees	61.29
Age		More than 50 employees	18.89
Under 30 years old	23.04	Age	
30–40 years old	48.38	<5 years	23.50
41–50 years old	17.97	5–10 years	38.25
51 years old and above	10.59	11–15 years	17.05
Years of experience		More than 15 years	21.19
Below 5 years	39.17		
5–10 years	40.09		
Above 10 years	20.73		

### Non-response bias, multicollinearity, and common method bias remedies

Following Armstrong and Overton (1977), we compared early respondents to late ones to assess the non-response bias. The *t*-test results showed that there were no statistically significant differences (*ρ <* 0.05 ) between the early and late responses, indicating that non-response bias is not a concern in this study.

Moreover, we used variance inflation factors (VIF) for each of the items to test the structural model for multicollinearity. Hair et al. (2018) suggested that the VIF cut-off value should be lower than 5.0 to mitigate multi-collinearity. All VIF values in the current study were between 1.295 and 2.831, which suggests that collinearity is not an issue.

Additionally, because we employed self-reported surveys, CMB might be a potential issue. Accordingly, we used the procedural remedies proposed by Podsakoff et al. (2003) to minimize CMB. First, we ensured respondents’ anonymity, separated the predictor and criterion variables to appear independent, and used well-prepared scales with established psychometric features to reduce social desirability bias. Second, we utilized SPSS to run the test of Harman’s single-factor (Podsakoff et al., 2003), which is adopted when a single variable explains the majority of the deviation. The entire variation for a single variable was found to be 35.359%, which is lower than 50%, indicating that CMB had no impact on our data.

### Questionnaire design and measures

In the current study, we employed mature measuring scales from earlier research. A five-point Likert scale was utilized to score each question on the survey.

#### Relational capital

The measurement of relational capital was derived from the study of Liao (2018), and included four items. A sample item is “when our SME faced difficulties amidst the pandemic, our partners were still ready to give support.” (see Appendix 1).

#### Environmental scanning

The environmental scanning measurement was mainly derived from Haarhaus and Liening (2020), and included four items. A sample item is “amidst the pandemic, our SME utilized different information sources while scanning the environment.” (see Appendix 1).

#### Organizational resilience

We assessed organizational resilience *via* a (9) item scale derived from Kantur and Iseri-Say (2015). This scale included three dimensions of robustness, agility, and integrity. The number of items used for robustness, agility, and integrity were four, three, and two, respectively. A sample item is “in the face of the pandemic, our SME stood straight and maintained its position.” (see Appendix 1).

## Data analysis and results

To conduct a partial least squares (PLS) analysis on the data, we used Smart PLS software, version 3.0. Since PLS–SEM has been extensively used in theory testing and validation, we employed the structural equation modeling (SEM) technique and partial least square (PLS). PLS–SEM is extensively adopted in numerous business fields as it provides robust estimations of the structural model, particularly for complicated structural models (Afthanorhan, 2013). Additionally, applying the PLS technique is feasible for the samples with relatively small sizes and particularly suitable for estimating inner and exterior model parameters and non-parameter bootstrapping with 5,000 replications (Hair et al., 2012). As recommended by Henseler et al. (2014), we used a two-step process that included: (1) evaluation of the measurement model; and (2) evaluation of the structural model.

### Assessment of measurement model

#### Assessing convergent validity

**Figure 2:**
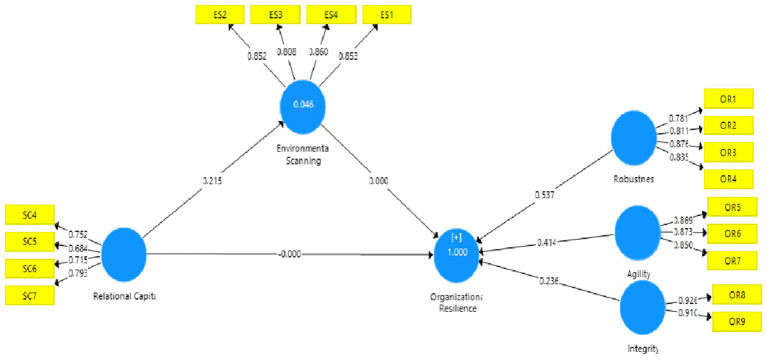
Factor loadings of individual items.

According to the suggestions of Hair et al. (2017), the validity and reliability of the items and latent constructs were evaluated. Each item has a high factor loading to its corresponding construct. Figure 2 shows that all factor loadings are between 0.684 and 0.926 indicating that they all are more than the proposed threshold of 0.5 (Hair et al., 2014). Besides, as indicated in Table 2, we evaluated latent constructs’ reliability through composite reliability (CR) and cronbach’s alpha (*α*) with a threshold at 0.70 (Hair et al., 2010). Both of the indicators of reliability are above 0.70 for all latent constructs (see Table 2). The assessment of convergent validity *via* average variance extracted (AVE) shows that all latent constructs above the 0.50 cutoff as suggested by Fornell and Larcker (1981) (see Table 2).

**Table 2:** Variables’ reliability and validity.

Measurement items	*α*	CR	AVE
Relational capital	0.719	0.826	0.543
Environmental scanning	0.865	0.908	0.712
Organizational resilience	0.881	0.905	0.516
Robustness	0.845	0.896	0.683
Agility	0.831	0.899	0.747
Integrity	0.814	0.915	0.843

#### Discriminant validity

To establish discriminant validity, we adopted individual items’ cross loadings as well as the Fornell-Larcker criterion and Heterotrait-Monotrait Ratio (HTMT).

##### Cross loadings of individual items

The extent to which a construct truly distinguishes itself from the other constructs according to empirical standards is referred to as discriminant validity. Therefore, each construct must be unique and reflect phenomena that are not addressed by the other constructs in the same model in order for discriminant validity to be established (Hair et al., 2014). In order to assess discriminant validity, we calculated individual items’ cross loadings. As shown by bold numbers for all items in Table 3, items must score higher on their own constructs within the model in order to attain discriminant validity (Fornell and Larcker, 1981; Chin, 1998).

**Table 3:** Cross loadings of individual items.

	Environmental scanning	Relational capital	Robustness	Agility	Integrity
ES1	**0.853**	0.191	0.281	0.237	0.137
ES2	**0.852**	0.194	0.248	0.286	0.128
ES3	**0.808**	0.125	0.253	0.356	0.096
ES4	**0.860**	0.211	0.318	0.328	0.114
RC1	0.196	**0.752**	0.353	0.226	0.354
RC2	0.064	**0.684**	0.335	0.209	0.339
RC3	0.216	**0.715**	0.222	0.303	0.278
RC4	0.151	**0.793**	0.312	0.358	0.394
OR1	0.229	0.370	**0.781**	0.466	0.424
OR2	0.314	0.353	**0.811**	0.642	0.337
OR3	0.260	0.362	**0.876**	0.503	0.413
OR4	0.278	0.286	**0.835**	0.531	0.344
OR5	0.260	0.349	0.594	**0.869**	0.339
OR6	0.340	0.321	0.536	**0.873**	0.316
OR7	0.330	0.302	0.553	**0.850**	0.401
OR8	0.179	0.438	0.450	0.398	**0.926**
OR9	0.075	0.415	0.389	0.349	**0.910**

The values in boldface represent score of each item on its own construct.

##### Fornell-Larcker criterion

Table 4 illustrates that all correlations were less than the square root of the average variance extracted (AVE) for each focal construct (Henseler et al., 2009), demonstrating that the scales utilized in our study are internally consistent and valid.

**Table 4:** Fornell-Larcker Criterion (correlation between constructs and the square root of AVE).

	Relational capital	Environmental scanning	Robustness	Agility	Integrity
Relational capital	**0.737**				
Environmental scanning	0.215	**0.844**			
Robustness	0.415	0.328	**0.827**		
Agility	0.375	0.358	0.650	**0.864**	
Integrity	0.465	0.141	0.458	0.408	**0.918**

The values in boldface represent square roots of AVE.

##### Heterotrait-Monotrait ratio

Table 5 illustrates the Heterotrait-Monotrait ratio (HTMT) results. The higher HTMT in this study is significantly less than the conservative level of 0.85 (Kline, 2011), with a value of 0.773, demonstrating discriminant validity.

**Table 5:** Heterotrait-Monotrait Ratio (HTMT).

	Agility	Environmental scanning	Integrity	Relational capital
Environmental scanning	0.423			
Integrity	0.494	0.165		
Relational capital	0.481	0.273	0.605	
Robustness	0.773	0.381	0.552	0.533

### Assessment of structural model

#### Assessing *R* square (*R*^2^)

The $R^{2}$ value is the proportion of variation in the dependent variable (s) that can be traced to one or more predictor variables (Hair et al., 2010). $R^{2}$ cut-off values of 0.67, 0.33, and 0.19, respectively, represent substantially strong, moderately strong, and weak values (Chin, 1998). According to $R^{2}$ calculation, relational capital and environmental scanning jointly explained 31% of the variance in organizational resilience.

#### Assessing *F* square (*f   *^2^)

When a specific predictor latent variable is omitted from the structural model, Chin (1998) advised assessing the change in $R^{2}$ and assessing whether the excluded variable has a substantial impact on the dependent variable. The *f *^2^ is the change in $R^{2}$ resulted from removing a single predictor latent variable.

Values ranging from 0.02 to 0.15 are used to indicate small effect sizes, 0.15 and 0.35 are used to represent medium impact sizes, and values above 0.35 are used to represent large effect sizes (Cohen, 1988). *f *^2^ values indicated that relational capital had a small effect on environmental scanning (0.048), and a medium effect on organizational resilience (0.269). Further, ES had a small effect on OR (0.092).

#### Assessing *Q* square (*Q*^2^)

Furthermore, we estimated *Q*^2^ using blindfolded in order to evaluate the structural model. In PLS–SEM, a *Q*^2^ value greater than zero for a specific endogenous construct demonstrates the path model’s predictive relevance for a particular dependent construct, and once the structural model exhibits this predictive relevance, it accurately predicts the data that are not considered in the model’s estimation (Hair et al., 2016). In the current study, the *Q*^2^ values of ES (0.031) and OR (0.155) are acceptable, indicating that the minimum requirements are fulfilled.

#### Hypotheses testing

We employed the Preacher and Hayes (2004, 2008) bootstrap approach to test the hypotheses. Relational capital’ direct effect on organizational resilience is 0.441, which is statistically significant (*p*-value = 0.000) based on *T* statistics > 1.96, value of *p* < 0.05 and the confidence interval of 0.321 to 0.549 does not contain zero, indicating that it is statistically different from zero. This means that H1 is accepted. The investigation of the mediating role of ES in the RC–OR relationship shows that RC significantly affects ES (*a* = 0.215, *p*-value = 0.002), while ES in turn significantly affects OR (*b* = 0.258, value of *p* = 0.000; see Table 6). The results further reveal that the indirect effect of RC on OR *via* ES (ab = 0.055, value of *p* = 0.008) is statistically different from zero, as indicated by a 95% bootstrap confidence interval of 0.018–0.099 (see Table 6). Hence, we find support for H4 that ES partially mediates the relationship between RC and OR relationship.

**Table 6:** Path coefficients for the different models.

Effects	Model 1 (without mediator)							
	Coefficient	SE	*T*-values	Values of *p*	2.5%	97.5%	Hypothesis	Result
RC → OR	0.441	0.062	7.162	0.000	0.321	0.549	H1	Accepted
RC → ES	0.215	0.068	3.165	0.002	0.090	0.355	H2	Accepted
ES → OR	0.258	0.073	3.538	0.000	0.104	0.402	H3	Accepted
	**Model 2 (with mediator)**							
Indirect RC → ES → OR	0.055	0.021	2.647	0.008	0.018	0.099	H4	Accepted

## Conclusion and discussion

The COVID-19 epidemic has significantly harmed enterprises and has devastated every community (Yu et al., 2022). Issues including shifts in international and export orders and a lack of raw materials have affected all industries, including the food and beverage industry (Aqeel et al., 2021; Li et al., 2022). Accordingly, academics began to focus their research on how companies can survive the pandemic. As a result, we investigated how Egyptian food and beverage SMEs remained resilient during the COVID-19 epidemic by investigating the impact of relational capital on organizational resilience both directly and indirectly *via* the mediation of environmental scanning of Egyptian food and beverage SMEs. Since research in this area is still relatively lacking, the current study is among the first to examine the factors that Egyptian food and beverage SMEs used to combat the COVID-19 epidemic.

It is argued that the food and beverage industry in Egypt is marked by weak connections between producers and processors, which results producing goods with inferior quality and packaging and has a detrimental impact on the trade balance and ultimate product prices (UNIDO, 2020). Therefore, in this study, we concentrated on investigating the role of building strong external relations with different business parties on the resilience of Egyptian food and beverage SMEs. In general, the results asserted that food and beverage SMEs who maintained strong relations with external stakeholders along with monitoring their external environments were the only survivals in the face of COVID-19.

The findings showed that ES positively affected OR (H3 was accepted). This result agrees with the notion of Akgün and Keskin (2014), McManus (2008), Hamel and Valikangas (2003) and Reeves et al. (2016) who emphasized that recognizing weak signs of environmental shifts is necessary for successful response and survival. Moreover, Vakilzadeh and Haase (2021) asserted that environmental scanning, as a predictive tool, is considered a building block of resilience. Besides, in accordance with our results, YahiaMarzouk and Jin (2022a) found that environmental scanning activities positively affected OR, namely robustness and agility.

Further, the results confirmed the positive impact of relational capital on organizational resilience (H1 was accepted). The extant research on relational capital and crisis situations in enterprises revealed that both of these topics have not been sufficiently investigated (Walecka, 2021). While research on the factors that influence an organization’s ability to withstand a crisis abounds in the literature on crisis management, it rarely emphasizes the significance of relational capital as a medium in anti-crisis strategies (Walecka, 2021). Accordingly, we came to the idea that relational capital is an important variable affecting the resilience of a company to the COVID-19 crisis, an idea that is still largely lacking amidst the COID-19 period, particularly within Egyptian food and beverage industry. Our study, therefore, is the first endeavor to empirically examine the role of relational capital in enhancing the resilience of Egyptian food and beverage SMEs to the COVID-19 pandemic. Our results, then, confirmed this expectation. More specifically, the result of H1 implies that the relational capital of SMEs contributes to their response to disturbances *via* facilitating the establishment of solutions for handling unexpected events and directing the collective effort towards finding mutually advantageous solutions (Gittell et al., 2006; Ortiz-de-Mandojana and Bansal, 2016; Ahangama et al., 2019). These ties inherent in RC connect different groups, allowing information flow among them, and generating more opportunities to deal with difficult circumstances. Therefore, in light of our results, we can conclude that RC could enhance the building of OR through utilizing and strengthening existing links, mutual transformation, and the generation of common situational perception (Sienkiewicz-Małyjurek, 2022).

Our result of H1 agrees with the results of Pawłowska (2018); Chowdhury et al. (2019); Jia et al. (2020); Sienkiewicz-Małyjurek (2022) who found that networks and resources enterprises can access through their RC help them develop organizational resilience. More importantly, as RC appears to be important for survival amidst crisis times, we conducted our study amidst the period of COVID-19 proved the significance of having strong relationships and networks with business partners to support OR. Similar to our investigation, Walecka (2021) found that the RC with external stakeholders of the firms surveyed in Poland enabled them to avoid several crises and improved their resilience to the COVID-19 crisis due to the right attitudes of their business parties.

Results from the Chinese context also agree with our results. In China, a similar result was justified through the impact of Confucian values on business relationships provided by prior research (Tan and Chee, 2005; Yan and Sorenson, 2005), which contend that mutual trust inherent in the interpersonal relationships count more than business ones in Chinese culture. As a result, the organizational and cultural contexts largely can also shape collaborative behaviors amidst crises.

Similar to China, Egypt is classified as a collectivist society on Hofstede’s individualism versus collectivism dimension. This classification therefore indicates that both nations have a propensity to prioritize group thinking or group conduct over individual thought. Managers typically work in groups in collectivist societies and strive to attain higher-order goals, just like in Egypt and China. Membership in or affiliation with groups such as business networks and prominent social circles can offer information and development opportunities with these values and within an organizational setting (Shaaban and Moneim, 2020). This similarity in the collectivism between Egypt and China highly explains why our results within the context of Egypt agree with those of Tan and Chee (2005); Yan and Sorenson (2005) within the Chinese context, since both countries are collectivistic ones that appreciate the belongings to in-groups (e.g., networks, organizations) who look after them in exchange for information and loyalty instead of looking after themselves and their immediate own benefits. Our results, therefore, may differ from those of studies within individualism countries such as North America and some other western nations, an interesting topic for future investigation.

An important contribution of our study pertains to the impact of relational capital on environmental scanning. Despite the large number of empirical research that have investigated ES activities in firms around the world (e.g., Jorosi, 2008; Wong and Hung, 2012; Aldehayyat, 2015), and the empirical studies that confirmed its positive impact on resilience and survival during crises (e. g., Vakilzadeh and Haase, 2021; YahiaMarzouk and Jin, 2022a,b), studies examining the factors that may facilitate the environmental scanning activities are still largely lacking. In this regard, we argued that RC enables enterprises to collaborate with other businesses, communities, and governmental agencies out of a sense of mutual respect and trust in order to gather important information. Relational capital is particularly significant for businesses that depend more heavily on external resources, such as those in the food and beverage industry (Fang et al., 2016). The result of H2 confirmed our expectation.

Therefore, we add to the existing literature on environmental scanning by clarifying that in order to facilitate ES activities, organizations should possess strong relationships with their business partners because with trustworthy relationships with business partners will prevent them from being hesitant to share information or discuss issues regarding potential environmental issues. In other words, we proved that RC could facilitate the implementation of ES activities through promoting a continuous flow of information from several external sources. This result is similar to the argument of Zahoor and Gerged (2021) who asserted that alliances with different parties including clients, suppliers and/or rivals allow an SME to acquire information and knowledge that can enable the establishment of solutions for social and environmental problems. Specifically, in consistence with our results, they proved that RC is useful for facilitating information exchange (Zahoor and Gerged, 2021), which has the potential to promote ES activities.

## Managerial implications

Our study shows that food and beverage SMEs can depend on their external relationships with business partners effectively to rapidly survive amid the epidemic. RC can provide d and important information and resources to meet the fluctuations in the external environment during the COVD-19 crisis. Food and beverage firms could also enhance and facilitate their environmental scanning activities through depending on their strong relations with different stakeholders. These strong networks can allow information to quickly flow within the organization to provide the new goods needed during a crisis. Besides, the COVID-19 pandemic posed new ways of working such as online purchasing and digital transformation. In this vein, external relationships might help in establishing partnerships with businesses that have complementary skills, such those who are able to increase the number of online delivery and purchasing options and increase client value. Therefore, we suggest that food and beverage SMEs improve and strengthen their channels of communication and relations with external stakeholders such as customers and suppliers as a way to enhance their alertness to environmental changes, thereby improving their resilience to those changes. Given the importance of customer insights to enterprises, it is crucial to disseminate the information gained from ES activities throughout the enterprise, but especially to the senior managers who will be tasked with putting the findings into practice.

Correspondingly, the results of our study suggest that environmental scanning activities can be advantageous for food and beverage SMEs. As a result, they must constantly keep an eye out for changes in the external environment to design plans that will let them build on their strengths, address their weaknesses, and assure compliance with new regulations. SMEs that can gather, evaluate, and act on environmental scanning results will be more successful in adapting to new changes and so increasing their resilience. It is consequently simpler to detect and take advantage of chances for transformation and adaptation in accordance with these new preferences when food and beverage SMEs regularly scan the external environment. This keeps them alert and responsive to changes in customer preferences.

## Limitations and recommendations for future research

Although the current study is the first to examine the factors (i.e., relational capital) affecting environmental scanning activities during the pandemic, the role of relational capital in enhancing resilience of Egyptian food and beverage SMEs, and the mediating role of ES in the relationship between RC and OR, it is not without specific limitations. First, we collected the data from managers in food and beverage organizations only. Future research should examine the same model within different types of industries to generalize the study findings. Furthermore, since cross-sectional data were employed in this study, future research on relationships during crises could find it beneficial to use a longitudinal design. Future research could evaluate the role of ES prior to the COVID-19 crisis to see if our conclusions remain applicable in non-crisis scenarios.

Finally, co-creation practices provide consumers, markets, upward (suppliers), and downward (customers) relationships with the chance to generate value *via* interactions. Partouche-Sebban et al. (2022) emphasized that co-creation positively affects organizational processes and practices and the quickness and quality of the decision-making in organizations Co-creation encourages organizations to develop new resources, thereby adapting and responding quickly to environmental changes and disruptions (Ju et al., 2021). Accordingly, we recommend for future studies to explore the role of co-creation in supporting the relational capital of organizations and/or the interactive effects of co-creation along with RC in underpinning ES and OR.

## Data availability statement

The raw data supporting the conclusions of this article will be made available by the authors, without undue reservation.

## Author contributions

YM: writing original draft, writing literature review, collecting data, conducting statistical analysis, and writing discussion and conclusion. JJ: reviewed and approved the final version, and supervised this research. All authors contributed to the article and approved the submitted version.

## Conflict of interest

The authors declare that the research was conducted in the absence of any commercial or financial relationships that could be construed as a potential conflict of interest.

## Publisher’s note

All claims expressed in this article are solely those of the authors and do not necessarily represent those of their affiliated organizations, or those of the publisher, the editors and the reviewers. Any product that may be evaluated in this article, or claim that may be made by its manufacturer, is not guaranteed or endorsed by the publisher.
