# Supplementary material for: Does relational capital matter to food and beverage SMEs’ resilience? The mediating role of environmental scanning
Source: Front Psychol. 2022 Oct 5;13:1033837. doi: 10.3389/fpsyg.2022.1033837 (PMC9581219; doi:10.3389/fpsyg.2022.1033837)
Supplement: Supplementary file 1 [file Data_Sheet_1.pdf]

Supplementary materials
Appendix 1
Scales

Table (A1) Relational Capital scale items					
Items	Strongly Disagree	Disagree	Neutral	Agree	Strongly Agree
**1.** Amidst the pandemic, regarding the process of cooperation between our SME and its partners, there was no tendency for either party to harm the other	1	2	3	4	5
**2.** Amidst the pandemic, our SME enjoyed a good reputation in the outside world.	1	2	3	4	5
**3.** When our SME faced difficulties amidst the pandemic, our partners remained ready to give support	1	2	3	4	5
**4.** When a department in our SME faced a trouble amidst the pandemic, other departments took the initiative to help.	1	2	3	4	5

Table (A2) Environmental scanning scale items					
Items	Strongly Disagree	Disagree	Neutral	Agree	Strongly Agree
1. Amidst the pandemic, our SME performed environmental scanning in many different areas (e.g. technology, economy, politics, social issues).	1	2	3	4	5
2. Amidst the pandemic, our SME utilized different information sources while scanning the environment.	1	2	3	4	5
3. Amidst the pandemic, our SME analyzed the environment with a very long-term perspective (min. 10 years).	1	2	3	4	5
4. Amidst the pandemic, our SME systematically monitored developments (e.g. trends, technologies) whose relevance for our business was not yet to evaluate.	1	2	3	4	5

**Table (A3)** Organizational resilience scale items					
Items	Strongly Disagree	Disagree	Neutral	Agree	Strongly Agree
**Robustness**					
1. In the face of the pandemic, our SME stood straight and maintained its position.	1	2	3	4	5
2. In the face of the pandemic, our SME was successful in generating diverse solutions.	1	2	3	4	5
3. In the face of the pandemic, our SME showed resistance to the end in order not to lose.	1	2	3	4	5
4. In the face of the pandemic, our SME did not give up and continued its path.	1	2	3	4	5
**Agility**					
5. In the face of the pandemic, our SME rapidly took action.	1	2	3	4	5
6. In the face of the pandemic, our SME developed alternatives in order to benefit from negative circumstances.	1	2	3	4	5
7. In the face of the pandemic, our SME was agile in taking required action when needed.	1	2	3	4	5
**Integrity**					
8. In the face of the pandemic, our SME was a place where all the employees engaged to do what is required from them.	1	2	3	4	5
9. In the face of the pandemic, our SME was successful in acting as a whole with all of its employees.	1	2	3	4	5
